# Factors associated with masticatory function as measured with the Mixing Ability Test in patients with head and neck cancer before and after treatment: a prospective cohort study

**DOI:** 10.1007/s00520-022-06867-0

**Published:** 2022-02-01

**Authors:** Jorine A. Vermaire, Cornelis P. J. Raaijmakers, Evelyn M. Monninkhof, Irma M. Verdonck-de Leeuw, Chris H. J. Terhaard, Caroline M. Speksnijder

**Affiliations:** 1 Department of Radiation Oncology, Imaging Division, University Medical Center Utrecht, Utrecht University, Utrecht, the Netherlands; 2 Department of Epidemiology, Julius Center for Health Sciences and Primary Care, University Medical Center Utrecht, Utrecht University, Utrecht, the Netherlands; 3 Department of Otolaryngology-Head and Neck Surgery and Cancer Center Amsterdam, Amsterdam UMC, Vrije Universiteit Amsterdam, Amsterdam, the Netherlands; 4 Department of Clinical, Neuro- and Developmental Psychology, Amsterdam Public Health Research Institute, Vrije Universiteit Amsterdam, Amsterdam, the Netherlands; 5 Department of Oral and Maxillofacial Surgery and Special Dental Care, University Medical Center Utrecht, Utrecht University, G05.122, P.O. Box 85.500, 3508 GA Utrecht, the Netherlands; 6 Department of Head and Neck Surgical Oncology, University Medical Center Utrecht, Utrecht University, Utrecht, the Netherlands

**Keywords:** Mixing Ability Test, Mastication, Linear mixed-effects model, Associative model, Head and neck cancer

## Abstract

**Purpose:**

After treatment for head and neck cancer (HNC), patients often experience major problems in masticatory function. The aim of this prospective cohort study among patients with HNC was to investigate which personal and clinical factors are associated with masticatory function from diagnosis up to 2 years after treatment with curative intent.

**Methods:**

Masticatory function was measured using the Mixing Ability Test (MAT) before treatment (baseline), and 3, 6, 12, and 24 months after treatment. A linear mixed-effects model with a random intercept and slope was conducted to investigate changes over time and the association with personal (sex, age) and clinical (tumor site, tumor stage, treatment modality) factors as measured at baseline.

**Result:**

One-hundred-twenty-five patients were included. The prevalence of masticatory dysfunction was estimated at 29% at M0, 38% at M3, 28% at M6, 26% at M12, and 36% at M24. A higher (worse) MAT score was associated with age, tumor stage, tumor site, timing of assessment, and the interaction between assessment moment and tumor site.

**Conclusion:**

In patients with HNC, masticatory function changed over time and dysfunction was associated with a higher age, a tumor in the oral cavity, a higher tumor stage, and a shorter time since treatment. The prevalence of masticatory dysfunction ranged from 26 to 38%.

**Supplementary Information:**

The online version contains supplementary material available at 10.1007/s00520-022-06867-0.

## Introduction

Following treatment for head and neck cancer (HNC), patients may experience major problems in masticatory function, which may lead to physical and emotional dysfunctioning as well [1].

Many factors can influence the masticatory process, such as dentition, bite force, amount and composition of saliva, and neuromuscular control of chewing and swallowing [2]. Treatment may result in deterioration of dentition and mastication, which can still be present 5 years after oncological intervention [3]. Deficiencies in masticatory function may lead to changes in diet, because some foods become troublesome to eat. Malnutrition may be associated with dysphagia, and can influence quality of life in those patients [4]. After treatment for HNC, the type of treatment results in different deficiencies in masticatory performance. Surgery can result in disabling alterations of functional components needed for occlusion, such as the mandible, temporomandibular joint (TMJ), muscles of mastication, or teeth [5]. Radiation therapy (RT) often mandates the extraction of teeth, which require replacement after treatment, often resulting in decreased masticatory function. In addition, radiation dose can affect the muscles of mastication and the TMJ by decreasing the range of motion of the mandible, resulting in a decreased mouth opening and restricting the size of the food bolus [5]. When salivary glands are included in the radiation field, varying degrees of xerostomia can be observed, which adversely affect the maintenance of teeth, and the formation and manipulation of the food bolus. Chemotherapy (CT) can cause mucositis, xerostomia, tooth loss, chewing difficulty, and neurotoxicity, which can restrict masticatory function as well [5, 6].

In order to reduce the risk of masticatory dysfunction before and after curative treatment for HNC, it is important to identify factors affecting masticatory performance. With the help of an associative model, patients in potential need of oral rehabilitation during or after treatment for HNC can be identified. Previous studies that focus on masticatory function, use trismus or patient reported outcomes as outcome measure, or investigate only a sub-group of patients (e.g. patients with oral cancer or patients treated with surgery) [3, 7–9]. To our knowledge, objective measures in patients with head and neck cancer and with different treatment modalities have not been performed yet. In addition, the course of masticatory function before and after treatment for patients with head and neck cancer has not been described. The aim of this prospective study was therefore to identify personal and clinical factors associated with objective masticatory function in patients with head and neck cancer before, and 3, 6, 12, and 24 months after treatment. In addition, the prevalence of masticatory dysfunction before and after treatment was assessed.

## Materials and methods

Patients were included by convenience sampling when they were 18 years or older, were diagnosed with oral, oropharyngeal, hypopharyngeal, or laryngeal cancer, and were treated with a curative intent at the University Medical Center Utrecht (UMCU), the Netherlands, between September 2014 and June 2018. Patients with recurrent or residual disease, cognitive impairments, and patients having trouble understanding or reading the Dutch language were excluded. All patients signed written informed consent before participation. The study protocol of this prospective cohort study was approved by the Medical Ethics Committee of the Netherlands (NL45051.029.13), and is part of the NET-QUBIC research [10]. Patient data about age, sex, tumor stage [11], tumor site, and treatment were collected. Patients were assessed before primary treatment (baseline, M0), and 3 (M3), 6 (M6), 12 (M12), and 24 months after treatment (M24). At every assessment, the Mixing Ability Test measuring masticatory performance was performed.

### Mixing Ability Test

The Mixing Ability Test (MAT) consists of two layers of wax, with the colors red and blue (Plasticine modelling wax, non-toxic DIN EN-71, art. nos. crimson 52,801 and blue 52,809, Stockmar, Kalten Kirchen, Germany) [3, 12–14]. The total thickness is 3 mm, with a diameter of 30 mm. The outcome variable is called the Mixing Ability Index (MAI), and ranges between 5 and 30, where a lower MAI score implies a better mixed tablet and better masticatory performance. A subject was asked to chew on this tablet 20 times in order to mix the two colors. The tablet was then flattened, pressed to a thickness of 2 mm, and scanned on both sides using a high quality scanner (Epson® V750, Long Beach, CA, USA). The scanned images were processed using Adobe Photoshop CS3 extended (Adobe, San Jose, CA, USA). The histograms of both sides of the flattened and scanned wax tablet were added to obtain red and blue intensity distributions. The spread of the color intensities was measured, and a mixing ability score was calculated [13]. In previous research, this test showed a good reliability (ICC = 0.886) when comparing test and retest [15]. To identify patients with masticatory dysfunction, a cut-off value was calculated, based on a value larger than 2 standard deviations from the mean value of healthy subjects, as calculated in previous research. A cut-off value of ≥ 20.5 indicated masticatory dysfunction [15].

### Statistical analyses

Descriptive statistics were used to describe the study population. A Kruskal–Wallis H test was performed to examine differences in age between different tumor sites, and a chi-square test was run to test for differences in sex, primary treatment, and tumor stage between tumor sites. A linear mixed-effects model (LMM) with the MAT as dependent outcome measure was conducted to investigate changes over time and the effect of patient characteristics and clinical parameters on MAT outcome [16]. Akaike’s Information Criterion (AIC) was used to select the most appropriate covariance structure to fit the data [17]. To account for within-patient correlations, a random patient factor was added, and a random intercept was used to account for different entry levels of patients. The fixed-effect factors tumor site, treatment modality, tumor stage, timing of assessment, sex, and age, as well as 2-way interactions of the factors tumor site, treatment modality, and tumor stage during the assessment period were assessed using the AR(1) method (first-order autoregressive covariance pattern) for parameter estimation. Tumor site consisted of 3 levels: oral cavity, oropharynx, and hypopharynx and larynx. Treatment modality consisted of 4 levels: RT, chemo radiation therapy (CRT), surgery, and a combination of surgery followed by post-operative RT or CRT. Tumor stage consisted of 4 levels (stage 1 to 4), timing of assessment consisted of 5 levels (M0, M3, M6, M12, and M24), sex consisted of 2 levels (male and female), and age was defined as a continuous variable. The model included a stepwise backward selection of factors, in which factors not significant at a *p* ≤ 0.10 level were removed, beginning with the interactions. A hierarchical structure was maintained, meaning that if an interaction was included in the model, the main effects were also represented in the model. Risk factors were reported as estimated unstandardized regression coefficients with 95% confidence intervals (CI) and p-values.

A score above the cut-off value of 20.5 was used to create a Receiver Operating Characteristic (ROC) curve, to help facilitate the use of the linear mixed-effects model in identifying factors associated with swallowing problems in patients with HNC.

The coefficients of the significant covariates, together with the value of the intercept of the mixed model analysis, were combined into a formula for the estimated MAT. The intercept is the value of the estimated MAT in which all coefficients remain zero. Addition of the coefficients will lead to an increase or decrease of the estimated MAT. For each time point, the formula was filled with average variable values for significant coefficients, as calculated by a restricted maximum likelihood approach (REML). Model assumptions were verified by plotting residuals versus fitted values. All analyses were performed using Statistical Package for the Social Sciences (SPSS) version 25 (Chicago, IL, USA). A p-value below 0.10 was considered statistically significant.

## Results

**Fig. 1:**
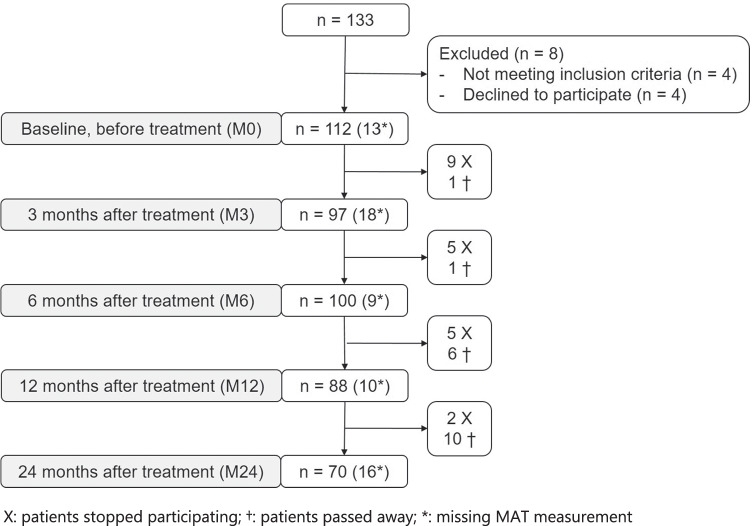
Flowchart depicting the number of patients at each time point

Baseline demographics and clinical characteristics are shown in Table 1 for the total patient group, and for subgroups based on tumor site. A total of 125 patients enrolled in this study, of which 112 underwent measurements at M0, 97 at M3, 100 at M6, 88 at M12, and 70 at M24 (Fig. 1). During a 2-year follow-up, 18 patients were deceased, and 21 patients dropped out. The mean MAT score was 18.8 (SD = 3.6) at M0, 19.2 (SD = 4.3) at M3, 19.0 (SD = 3.6) at M6, 18.3 (SD = 4.0) at M12, and 18.8 (SD = 3.7) at M24. The number of patients with masticatory dysfunction (a value above the MAT cut-off score of ≥ 20.5) was 32 at M0 (29%), 37 at M3 (38%), 28 at M6 (28%), 23 at M12 (26%), and 25 at M24 (36%).

**Table 1:** Baseline characteristics of patients with HNC that performed the MAT based on all patients, and subgroups of patients based on tumor site

Variable		Tumor site			*p*-value
	All patients	Oropharynx (*n* = 48)	Larynx and hypopharynx (*n* = 42)	Oral cavity (*n* = 35)	
	median (IQR)	median (IQR)	median (IQR)	median (IQR)	
Age	63.0 (15.0)	59.0 (14.5)	64 (12.5)	64 (18)	0.142†
Sex	*n* (%)	*n* (%)	*n* (%)	*n* (%)	0.030*‡
* Male*	97 (77.6)	36 (75.0)	38 (90.5)	23 (65.7)	
* Female*	28 (22.4)	12 (25.0)	4 (9.5)	12 (34.3)	
Primary treatment					< 0.001*‡
* RT*	55 (44.0)	23 (47.9)	31 (73.8)	1 (2.9)	
* CRT*	31 (24.8)	24 (50.0)	6 (14.3)	1 (2.9)	
* Surgery*	25 (20.0)	1 (2.1)	4 (9.5)	20 (57.1)	
* Surgery with (C)RT*	14 (11.2)	0	1 (2.4)	13 (37.1)	
Tumor stage					0.001*‡
* I*	34 (27.2)	3 (6.3)	18 (42.8)	13 (37.1)	
* II*	26 (20.8)	10 (20.8)	7 (16.7)	9 (25.7)	
* III*	15 (12.0)	6 (12.5)	6 (14.3)	3 (8.6)	
* IV*	50 (40.0)	29 (60.4)	11 (26.2)	10 (28.6)	
Tumor site					
* Oropharynx*	48 (38.4)				
* Larynx and Hypopharynx*	42 (33.6)				
* Oral cavity*	35 (28.0)				

*CRT*: Chemo radiation therapy, *IQR*: Interquartile range, *n*: number of patients, *RT*: Radiation therapy

^*^:*p* ≤ 0.05, †:Kruskal–Wallis H test, ‡:chi-square test

**Fig. 2:**
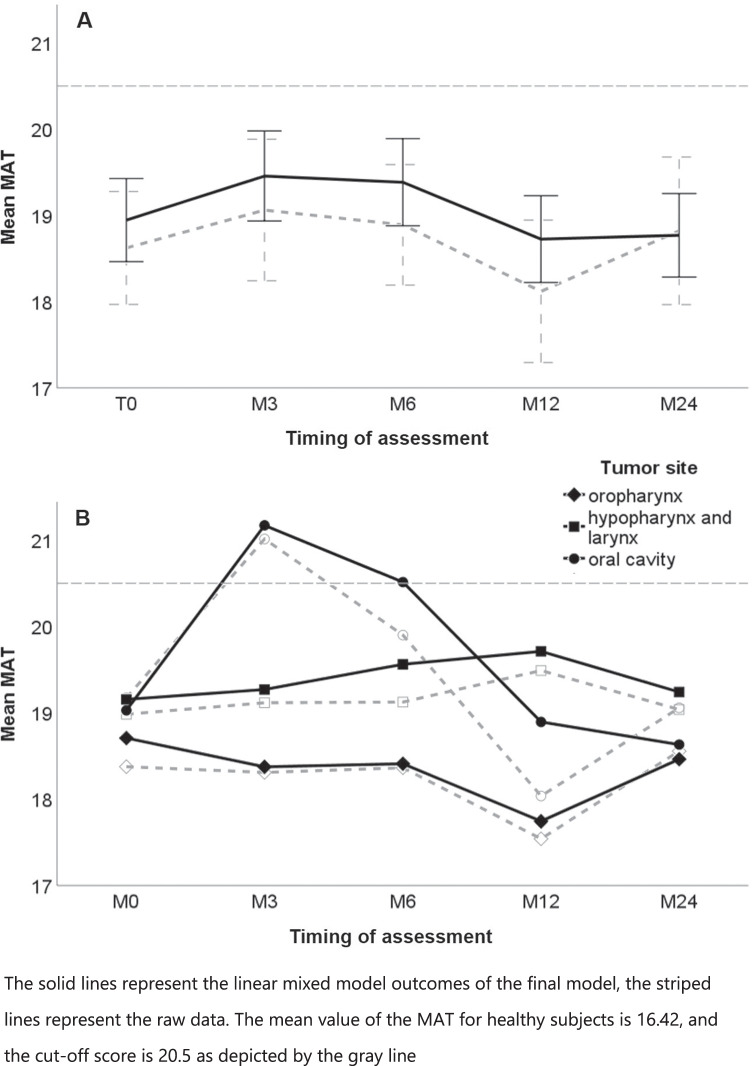
The mean MAT outcome for all patients with corresponding confidence intervals (**A**) and for patients based on tumor site (**B**)

LMM analysis showed that the MAT score increased 3 and 6 months after treatment, indicating a worse masticatory function. The MAT returned to baseline values 12 and 24 months after treatment (Fig. 2a). Sex was not associated with the MAT score, and was therefore removed from the model. The MAT score was associated with age, tumor stage, tumor site, and timing of assessment, and the interaction between timing of assessment and tumor site appeared of importance (Table 2). With increasing age, the MAT score increased as well (+ 0.08 each year, p-value = 0.008). Patients with tumor stage 1 and 2 had a lower MAT score in comparison to patients with stage 4 tumors (MAT score =  − 2.63, p-value = 0.001 and MAT score =  − 1.97, p-value = 0.018, respectively). After treatment, the MAT score increased with 2.14 (M3) (*p*-value =  < 0.001) and 1.49 (M6) (*p*-value = 0.014), and returned to baseline 1 year after treatment. The longitudinal course of MAT differed between tumor sites (Fig. 2b). The cut-off score was used to develop a ROC curve indicating masticatory dysfunction before and after treatment in patients with HNC (Appendix). The formula for the estimated MAT that was retained in the final model is shown in the footnote of Table 2.

**Table 2:** Linear mixed model estimates for the MAT

		Estimate (95% CI)	*p*-value	Interactions with timing of assessment									
*Intercept*		15.40											
				*M0*		*M3*		*M6*		*M12*		*M24*	
				Estimate (95% CI)	*p*-value	Estimate (95% CI)	*p*-value	Estimate (95% CI)	*p*-value	Estimate (95% CI)	*p*-value	Estimate (95% CI)	*p*-value
*Age*		0.08 (0.02 to 0.14)	.008*										
*Tumor stage*	*1*	− 2.63 (− 4.23 to − 1.03)	.001*										
	*2*	− 1.97 (− 3.59 to − 0.35)	.018*										
	*3*	0.25 (− 1.73 to 2.24)	.801										
	*4*	*Reference*											
*Timing of assessment*	*M0*	*Reference*											
	*M3*	2.14 (1.08 to 3.21)	< .001*										
	*M6*	1.49 (0.31 to 2.67)	.014*										
	*M12*	− 0.13 (− 1.45 to 1.18)	.840										
	*M24*	− 0.40 (− 1.94 to 1.15)	.613										
*Tumor site*	*Oral cavity*	*Reference*		*Reference*									
	*Oropharynx*	− 1.21 (− 2.94 to 0.51)	.166	*Reference*		− 2.48 (− 3.80 to -1.15)	< .001*	− 1.78 (− 3.29 to − 0.28)	.020*	− 0.83 (− 2.49 to 0.83)	.326	0.15 (− 1.74 to 2.04)	.874
	*Hypopharynx and Larynx*	− 0.13 (− 1.87 to 1.60)	.878	*Reference*		− 2.03 (− 3.45 to -0.61)	.005*	− 1.08 (− 2.67 to 0.50)	.181	0.69 (− 1.06 to 2.44)	.438	0.48 (− 1.51 to 2.47)	.634

^*^: *p* ≤ 0.10; SE: Standard Error

The following formula for the estimated MAT shows the significant variables and their coefficients that were retained in the model. Factors with a coefficient above 0 will increase the MAT outcome and therefore indicate a worse masticatory performance:

Estimated MAT = 15.40 + 0.08age—2.63tumorstage1—1.97tumorstage2 + 0.25tumorstage3 + 2.14M3 + 1.49M6—0.13M12—0.40M24—1.21oropharynx – 0.13hypopharynx&larynx – 2.48oropharynx*M3 – 2.03hypopharynx&larynx*M3 – 1.78oropharynx*M6 – 1.08hypopharynx&larynx*M6 – 0.83oropharynx*M12 + 0.69hypopharynx&larynx*M12 + 0.15oropharynx*M24 + 0.48hypopharynx&larynx*M24

## Discussion

This 2-year prospective study showed that the prevalence of masticatory dysfunction among patients with HNC was estimated at 29% before treatment, 38% at 3 months after treatment, 28% at 6 months, 26% at 12 months, and 36% at 24 months. The mean MAT values indicate a decrease in masticatory function 3 and 6 months after treatment, and a return to baseline values 1 and 2 years after treatment. Masticatory function was associated with age, tumor stage, tumor site, timing of assessment, and the interaction between tumor site and timing of assessment. The masticatory performance decreased with age. Furthermore, a higher tumor stage was associated with a worse masticatory performance. Patients with oral cavity tumors performed worse in comparison to those with oropharynx and hypopharynx and larynx tumors. Masticatory function worsened in patients with an oral cavity tumor from diagnosis up to 6 months after treatment, and returned to baseline levels 1 and 2 years after treatment. Patients with an oropharynx, hypopharynx or larynx tumor did not show this decrease in function after treatment.

### Comparison with literature

The association between age and worse masticatory function is found in previous research as well [18, 19]. It was suggested that this association is caused by different mechanisms: fewer contacts between functional units (for example caused by a lower number of teeth), the presence of xerostomia, and/or decreased oral muscle activities [20]. When patients lose their teeth, it is advised to install a suitable dental prosthesis, and to train and exercise the masticatory muscles in order to increase oral motor and sensory functions that are used in mastication [21]. In future research, it is therefore important to measure the number of teeth and number of occlusal units and include these as factors in the LMM.

Previous research on masticatory function as measured with the MAT focused on patients that received surgery for oral cancer, in which measurements were performed before surgery, 4–6 weeks after surgery, 6 months after surgery, and 1 and 5 years after surgery. Masticatory function worsened from baseline to 1 year after treatment, and recovered 5 years after treatment. These changes over time are in line with the results found in this study for patients with oral cancer. Other research in patients with oral cancer found that surgery and surgery followed by RT had a significant impact on oral function, and the recovery was less prominent in patients that received surgery followed by RT in comparison to patients that received surgery only. This was caused by the fact that patients treated with surgery and RT had larger tumors, more extended resections, and received RT which caused more symptoms [3]. Other research mainly focused on limited mouth opening (trismus) as outcome measure, which is also correlated to mastication [22]. It was found that trismus is significantly related to tumor stage, the use of RT and the use of free tissue reconstruction. Patients with stage 3 and 4 tumors, and patients receiving RT or a reconstruction had a smaller mouth opening [22]. The relation between chewing function and stage 4 tumors was described previously as well [23]. These risk factors are in line with the results found in this research, except for choice of treatment, which was not found in this study.

### Strengths and limitations

Strengths of our study were the prospective study design, the use of the LMM checklist with recommendations for reporting multilevel data and analyses [24], and the high test–retest reliability of the MAT as found in previous research [15]. Limitations were the low number of patients at follow-up, which limited the number of factors that could be explored with the LMM, and the relative large drop-out and missing values. These missing data might have affected the analyses, because it is unknown how these patients would have performed on the MAT. Although the LMM is better at handling missing values in comparison to other regression analyses, these regression models do not take into account the number of deaths as competing risk [25].

Although no significant correlations were found between the factors used in the LMM, treatment and tumor stage did differ between different tumor sites, as seen in Table 1: Patients with an oropharynx tumor most often received RT or CRT, while patients with an oral cavity tumor most often received surgery or surgery followed by RT or CRT. In addition, oropharynx tumors were most often stage 4 tumors, while hypopharynx and larynx tumors were most often stage 1 tumors. Therefore, the association found between MAT outcome and tumor site is, to a lesser extent, also caused by treatment modality and tumor stage. Because of the low number of patients in this study, no interactions between treatment, tumor stage and tumor site could be explored in the LMM.

The mean values indicate a decrease in masticatory function especially 3 and 6 months after treatment, and a return to baseline at 12 and 24 months after treatment. However, the cut-off values indicate masticatory dysfunction especially 3 and 24 months after treatment. Impairment after treatment varies greatly between patients; it is affected by site and extent of the tumor, age, irradiation site and dose, extent of tumor resection, and reconstruction procedures [26]. Acute toxicity after treatment (e.g. mucositis, xerostomia, tooth loss) causes a decrease in masticatory function, which slowly recovers over time. However, long term treatment effects may persist even beyond 5 years after treatment [27], which may explain the masticatory dysfunction of 36% at 2 years after treatment. Although an effort has been made to make a distinction based on tumor site, tumor stage, age, and treatment, future research should aim to investigate the discrepancy between mean values and cut-off values, and why more patients had problems 2 years after treatment in comparison to 1 year after treatment (based on the cut-off value), and why this does not translate to the mean values.

Previous research showed that the objective MAT and subjective patient reported outcomes related to mastication have a low correlation and can therefore not be used interchangeably [28]. A future study might aim at developing a prediction model with subjective outcomes, to study whether factors found in the current study would be the same when subjective measures are used. A recommendation would be to include a larger study group, to be able to include a larger number of potential predictors in the LMM and thus provide more reliable and focused results.

In conclusion, masticatory function can be influenced by treatment for head and neck cancer. Masticatory dysfunction was associated with a greater age, a tumor in the oral cavity, a higher tumor stage, and a shorter time since treatment. The prevalence of masticatory dysfunction ranged from 26 to 38% before and after treatment. It is important to identify patients at risk for developing masticatory problems, to inform them about possible problems that may occur during and after treatment, and to increase awareness about possibilities for patients regarding rehabilitation.

## Supplementary Information

Below is the link to the electronic supplementary material.

Supplementary file1 (PDF 201 KB)

## Data Availability

The collection and integration of large amounts of personal, biological, genetic and diagnostic information precludes open access to the NET-QUBIC research data. In the section Data and sample dissemination (www.kubusproject.nl) is described how the data are made available for the research community.
