# Supplementary material for: Factors associated with masticatory function as measured with the Mixing Ability Test in patients with head and neck cancer before and after treatment: a prospective cohort study
Source: Support Care Cancer. 2022 Feb 1;30(5):4429–36. doi: 10.1007/s00520-022-06867-0 (PMC8942951; doi:10.1007/s00520-022-06867-0)
Supplement: Supplementary file 1 — Supplementary file1 (PDF 201 KB) [file 520_2022_6867_MOESM1_ESM.pdf]

Factors associated with masticatory function as measured with the Mixing Ability Test in patients with head and neck cancer before and after treatment: a prospective cohort study

Supportive care in cancer

Jorine A. Vermaire, Cornelis P.J. Raaijmakers, Evelyn M. Monninkhof, Irma M. Verdonck-de Leeuw, Chris H.J. Terhaard, Caroline M. Speksnijder

Corresponding author

Caroline M. Speksnijder

Department of Oral and Maxillofacial Surgery and Special Dental Care

University Medical Center Utrecht, G05.122

P.O. Box 85.500

3508 GA Utrecht

The Netherlands

e-mail: C.M.Speksnijder@umcutrecht.nl

Appendix 1. Receiver operating characteristic (ROC) curve for mastication problems after treatment for head and neck cancer, using the linear mixed model

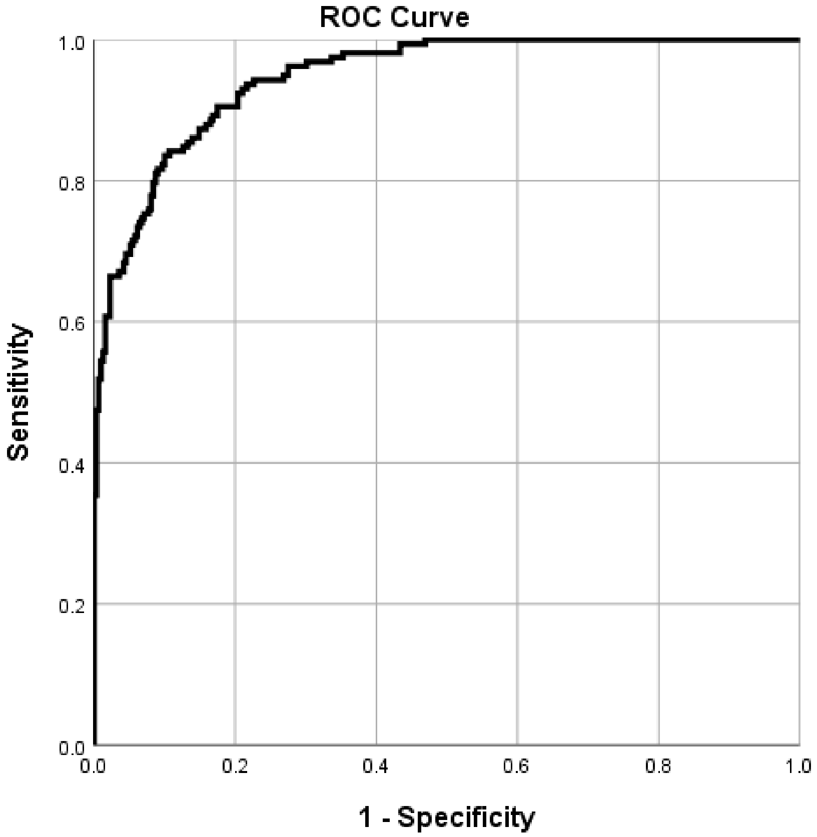

The area under the curve (AUC) is 0.947. The AUC at the different assessment moments is 0.935 (T0), 0.951 (M3), 0.967 (M6), 0.948 (M12), and 0.953 (M24). The AUC can vary between 0 and 1, where a value of 0 indicated that the model has no diagnostic power, and a value of 1 indicates that the model has a perfect diagnostic accuracy
